# Dynamic Transcriptome Profiling Reveals LncRNA-Centred Regulatory Networks in the Modulation of Pluripotency

**DOI:** 10.3389/fcell.2022.880674

**Published:** 2022-05-11

**Authors:** Shen Wang, Jun Zhang, Yu’an Ding, Haotian Zhang, Xiang Wu, Lingci Huang, Junjie He, Jun Zhou, Xiao-Min Liu

**Affiliations:** ^1^ School of Life Science and Technology, China Pharmaceutical University, Nanjing, China; ^2^ Key Laboratory of Pathogen Biology of Jiangsu Province, Nanjing, China

**Keywords:** lncRNA, m^6^A modificaiton, embryonic stem cell, Gm2379, cell fate

## Abstract

Long noncoding RNAs (lncRNAs) have emerged as vital regulators of gene expression during embryonic stem cell (ESC) self-renewal and differentiation. Here, we systemically analyzed the differentially regulated lncRNAs during ESC-derived cardiomyocyte (CM) differentiation. We established a perspicuous profile of lncRNA expression at four critical developmental stages and found that the differentially expressed lncRNAs were grouped into six distinct clusters. The cluster with specific expression in ESC enriches the largest number of lncRNAs. Investigation of lncRNA-protein interaction network revealed that they are not only controlled by classic key transcription factors, but also modulated by epigenetic and epitranscriptomic factors including N^6^-methyladenosine (m^6^A) effector machineries. A detailed inspection revealed that 28 out of 385 lncRNAs were modified by methylation as well as directly recruited by the nuclear m^6^A reader protein Ythdc1. Unlike other 27 non-coding transcripts, the ESC-specific lncRNA *Gm2379*, located in both nucleus and cytoplasm, becomes dramatically upregulated in response to the depletion of m^6^A or Ythdc1. Consistent with the role of m^6^A in cell fate regulation, depletion of *Gm2379* results in dysregulated expressions of pluripotent genes and crucial genes required for the formation of three germ layers. Collectively, our study provides a foundation for understanding the dynamic regulation of lncRNA transcriptomes during ESC differentiation and identifies the interplay between epitranscriptomic modification and key lncRNAs in the regulation of cell fate decision.

## Introduction

Embryonic stem cells (ESCs) are pluripotent stem cells derived from the inner cell mass of the blastocyst. They possess dual ability to self-renew unlimitedly and differentiate into multiple tissue types found in the adult animal. Both ESC proliferation and differentiation are complex processes requiring the synergetic regulation by transcription factors (TFs), epigenetic factors, histone modification and chromatin remodelers (Martello and Smith, 2014; Atlasi and Stunnenberg, 2017; Chen et al., 2017; Zhao and Jin, 2017). Apart from protein-mediated transcriptional and epigenetic networks, long non-coding RNAs (lncRNAs) emerge as new players which guide the fate regulation of pluripotency and stem cell differentiation (Fico et al., 2019; Chen et al., 2020; Constanty and Shkumatava, 2021). They are a major group of non-coding RNAs with over 200 nucleotides in length but generally lack of translated open reading frames and obvious protein coding capacity (Statello et al., 2021). Many lncRNAs are dynamically regulated during ESC differentiation and expressed highly at specific developmental stage (Luo et al., 2016; Li et al., 2017). Therefore, establishment of lncRNA expression profiles at different developmental stages will facilitate our understanding of gene regulatory events during normal development at additional layers.

Distinct from coding mRNAs, lncRNAs themselves serve as the functional unit to modulate the expression of target genes depending on their subcellular localization and interacting molecules (Carlevaro-Fita and Johnson, 2019; Yao et al., 2019). In the cytoplasm, lncRNAs generally function to regulate mRNA stability, mediate mRNA translation and act as competing endogenous RNAs (Statello et al., 2021). In comparison, the functional roles of nucleus-localized lncRNAs have been more extensively studied. The specific interacting proteins present in the nucleus endow lncRNAs with modulation of chromatin architecture and transcriptional activity (Bergmann and Spector, 2014; Rutenberg-Schoenberg et al., 2016; Statello et al., 2021). For example, *XIST* lncRNA is able to recruit the polycomb repressive complex (PRC) to trigger the PRC1-guided mono-ubiquitylation of histone H2AK119 and the PRC2-dependent H3K27me3, resulting in silencing of gene transcription (Plath et al., 2003; Fang et al., 2004). In addition, nuclear lncRNA also participate in establishment of the nuclear compartment organization via scaffolding. For example, *MALAT1* lncRNA functions as a scaffold molecule in nuclear speckles to regulate splicing *via* recruiting serine/arginine (SR) splicing factors (Tripathi et al., 2010). Thus, investigation of the interactions between lncRNAs with RNA-binding proteins (RBPs) are crucial for deciphering their functional roles in diverse biological processes.

In addition to the structural features, the modifications embedded in non-coding RNAs are also able to regulate target gene expression through recruiting a subgroup of specific RBPs (Liu et al., 2020, 2021; Chen et al., 2021; Lee et al., 2021; Wei and He, 2021). N^6^-methyladenosine (m^6^A), the most abundant modification identified in mRNA, has been recently found to exist on lncRNAs and associate with specific “reader” proteins to modulate their functionality. m^6^A residues on the *XIST* are able to recruit nuclear RBP YTH domain containing 1 (YTHDC1) and required for *XIST*-mediated transcriptional gene silencing (Patil et al., 2016). Besides, the interaction of YTHDC1 with methylated adenosines on MALAT1 has been identified to regulate homeostasis of nuclear speckles and cancer metastasis (Wang et al., 2021). During heat shock stress response, a novel lncRNA *Heat* silences stress gene expression via the m^6^A-YTHDC1 association (Ji et al., 2021). Despite the emergence of certain studies for m^6^A-guided lncRNA functionality, how the interplay between m^6^A and lncRNA regulate stem cell pluripotency and differentiation remains largely unknown.

In this study, we systematically analyzed the lncRNA expression at different stages during ESC-derived cardiomyocyte (CM) differentiation. We found that the largest group of lncRNAs enriched was expressed specifically in ESC. They were not merely modulated by cell fate-associated TFs, but also able to recruit RNA m^6^A epigenetic factors. Indeed, a subgroup of these lncRNAs were obviously modified by m^6^A and bound with the reader protein YTHDC1. Intriguingly, only one ESC-specific lncRNA *Gm2379* was sensitive to the dysfunction of m^6^A-regulatory proteins. Depletion of m^6^A-modified *Gm2379* resulted in dysregulated expressions of crucial genes required for ESC pluripotency maintenance and differentiation.

## Materials and Methods

### Cell Lines and Reagents

J-1 male mouse embryonic stem cells (ESCs) were purchased from the Cell Bank of Chinese Academy of Sciences. Wild-type and *Mettl3-*KO J-1 mouse ESCs were provided by Howard Y. Chang in Stanford University (Batista et al., 2014). *Ythdc1* ^flox/flox^ (Control) and *Ythdc1*-cKO (*Ythdc1-*KO) mouse ESCs were provided by Yawei Gao in Tongji University (Chen et al., 2021). Mycoplasma testing was performed routinely and cells tested negative were used in further experiments. Tissue culture plates were initially treated with 0.2% gelatin for at least 30 min at 37°C. ESCs were subsequently grown in gelatinized plates containing 15% KnockOut Serum Replacement (Gibco), 1% non-essential amino acids (Gibco), 1% Glutamax (Gibco), 1% Penn-Strep (Life technologies), 0.1 mM β-mercaptoethanol (Invitrogen) and 1000 U/ml ESGRO Recombinant Mouse leukemia inhibitory factor (LIF) Protein (Millipore).

### Cardiomyocyte Differentiation

To generate embryoid bodies (EBs), suspension of each cell line at a concentration of 10 × 10^4^cells per milliliter in differentiating medium (Knock-out DMEM supplemented with 15% FBS (Gibco), 1% non-essential amino acids, 1% Glutamax, 1% Penn-Strep, 0.1 mm β-mercaptoethanol) was deposited in 20 μL hanging drops in Petri dishes. After 2 days, the suspension-cultured cells were plated onto 0.2% gelatin-coated dishes in cardiomyocyte differentiation media (CMD) containing DMEM (BI), 15% FBS (Gibco), 1% Penn-Strep, 1% non-essential amino acids (Gibco), 1% Glutamine (Gibco), 0.1 mm β-mercaptoethanol and 1 mm Ascorbic Acid (Sigma) for continued differentiation. Cell culture media was changed every other day and contracting patches of cells were collected at indicated time points.

### Targeted Knockdown of *Gm2379* Long Noncoding RNAs in Mouse Embryonic Stem Cells

The shRNA targeting sequences were cloned into pRSI9-U6-(sh)-UbiC-TagRFP-2A-Blasticidin. Lentiviral particles were packaged using Lenti-X 293T cells. Virus-containing supernatants were collected at 48 h after transfection and filtered to eliminate cell debris. Wild-type and *Mettl3-*KO mESCs were infected by the lentivirus for 48 h and stable mESC knockdown lines were generated using blasticidin (MCE) selection (10 µg/ml). shRNA targeting sequences are listed below.

*Gm2379* (target sequence 1): 5′- GCA​CGG​CAT​TAC​AGA​ATT​TCA-3′; *Gm2379* (target sequence 3): 5′- GAA​AGC​CTT​CAT​AGG​AAA​CAG-3′; Scramble Target Sequence: 5′- AAT​TCT​CCG​AAC​GTG​TCA​CGT-3′.

### Real-Time Quantitative PCR

Total RNA from different cells was isolated from PBS-washed cells using Trizol reagent (Invitrogen). Isolated RNAs were used as templates for reverse transcription based on the manual for HiScript III 1st Strand cDNA Synthesis Kit plus gDNA wiper (Vazyme Biotech). Real-time PCR analysis was conducted with ChamQ SYBR qPCR Master Mix (Vazyme Biotech) and carried on a QuantStudio three Real-Time PCR System (Applied Biosystems). Primers for amplifying each target were listed in Supplementary Table S1.

### Cell Fractionation Assay

ESCs were pelleted by centrifugation at 500 g for 3 min at 4°C. After washing with phosphate-buffered saline (PBS) buffer twice, cells were lysed in ice-cold cytoplasmic lysis buffer (10 mM HEPES pH 7.5, 0.05%TritonX-100, 5 mM MgCl_2_, 10 U SUPERase-In and 1x protease inhibitor cocktail) on ice for 5 min. Samples were centrifuged at 2,000 g at 4°C for 10 min. The supernatant was collected as cytoplasmic fraction. The remaining nuclear pellets were washed with PBS twice and centrifuged at 2,000 g at 4°C for 2 min. After removing the supernatant, the nuclear pellet was resuspended in RIPA buffer (50 mM Tris-HCl, pH7.5, 150 mM NaCl, 1% NP-40, 0.5% sodium deoxycholate, 10 U SUPERase-In and 1 × protease inhibitor cocktail). This sample was collected as nuclear fraction. RNA from different fractions was isolated using TRIzol reagent (Invirtrogen), and the protein sample was prepared by adding SDS loading buffer.

### RNA-Seq

Total RNA was isolated using TRIzol reagent (Invitrogen). Ribosomal RNAs depletion, strand-specific library preparation and sequencing were performed at the Annoroad Gene Technology (Beijing, China). Two biological replicates were prepared for each differentiation phases. The libraries were sequenced on an Illumina Novaseq 6,000 system using the 2 × 150-bp paired-end read mode.

### Long Noncoding RNA-Seq Data Processing

The lncRNA-seq data has been deposited in NCBI’s Gene Expression Omnibus and is accessible through GEO Series accession number GSE197205. Trim Galore (v.0.6.6) was used to trim the low quality sequences (Martin, 2011). The paired-end reads were aligned to mouse genome (GRCm38) with HISAT2 aligner (v2.2.1) with --rna-strandness RF parameters and Ensemble genes (GRCm38.102) transcriptome annotation (Pertea et al., 2016). Then gene expression were quantified using featureCounts (v2.0.1) with -s 2 parameter (Liao et al., 2014), and differentially expressed genes were identifified using DESeq2 (v1.34.0) R package (Love et al., 2014). The FoldChange > 2 or < 0.5 and ajusted *p*-value < 0.05 genes were considered as differentially expressed genes. The TPM (Transcripts Per Kilobase of exonmodel per Million mapped reads) was used to produce heatmap.

### Fuzzy C-Means Clustering

LncRNAs with average TPM expressions in replicates from four differentiation stages were grouped into six different clusters using Mfuzz (v2.54.0) package in R with fuzzy c-means algorithm (Kumar and Futschik, 2007).

### Regulatory Motif and Functional Analysis of Embryonic Stem Cells-specific Long Noncoding RNAs

HOMER’s findMotifs.pl program was used to identify motifs in the promoters of lncRNA genes (encompassing regions 2000 bp upstream and 500 bp downstream of the transcription start site) from each cluster. If one lncRNA has multiple transcript isoforms, the longest transcript isoform was used for analysis and bedtools getfasta (v2.29.2) was used to extract sequnences. Enrichment analysis was performed on Gene Ontology website (Ashburner et al., 2000). Network plot was produced by Cytoscape (v3.8.2) (Paul Shannon et al., 1971).

### MeRIP-Seq and YTH Domain Containing 1 RIP-Seq Data Analysis

MeRIP-seq and RIP-seq datasets were obtained from the Gene Expression Omnibus (GEO) public database under accession number GSE145315 and GSE157265, respectively (Chen et al., 2021; Liu et al., 2021). Quality control of MeRIP-seq and RIP-seq data were performed using FastQC (v.0.11.9). Low-quality bases and adaptor contamination were removed by Trim Galore. Remaining reads were aligned to mouse genome version GRCm38 with HISAT2 aligner with default parameters. For RIP-seq data SAMtools (v1.13) was used to remove the reads with mapping quality below 30 (Li et al., 2009). m^6^A peaks and differential m^6^A peaks were identified using exomePeak2 (v1.6.1) R package with *p*_cutoff = 0.00001 and log2FC_cutoff = 1 parameters (Meng et al., 2014). Log_2_FoldChange < −1 and ajusted *p* value < 0.05 was considered as significantly downregulated peaks. For biological replicates, bedtools (v2.29.2) was used to intersect peaks with overlaped proportion > 0.5 (Meng et al., 2014). Peaks were annotated by HOMER’s annotatePeaks.pl program. The distribution of m6A peaks was plotted by R package Guitar (v.1.5.0) (Cui et al., 2016). The final bam files were transformed to bigwig format by using Deeptools bamCoverage (v3.5.1) with --normalizeUsing RPKM parameters (Ramírez et al., 2014). IGV (Integrative genomics viewer, v2.11.1) was used to visualize the aligned data (Thorvaldsdóttir et al., 2013). DESeq2 was used to identify Ythdc1 binding genes. The FoldChange > 2 and ajusted *p*-value < 0.05 genes were considered as Ythdc1 binding RNAs.

### RNA-Seq Data Analysis

*Ythdc1*‐KO and *Mettl3*‐KO RNA-seq datasets were obtained from the Gene Expression Omnibus (GEO) public database under accession number GSE157262. The expression analysis was performed based on the available processed fpkm-matrix files in the database.

## Results

### Dynamic Long Noncoding RNA Transcriptome During Embryonic Stem Cells-Derived Cardiac Differentiation

**FIGURE 1:**
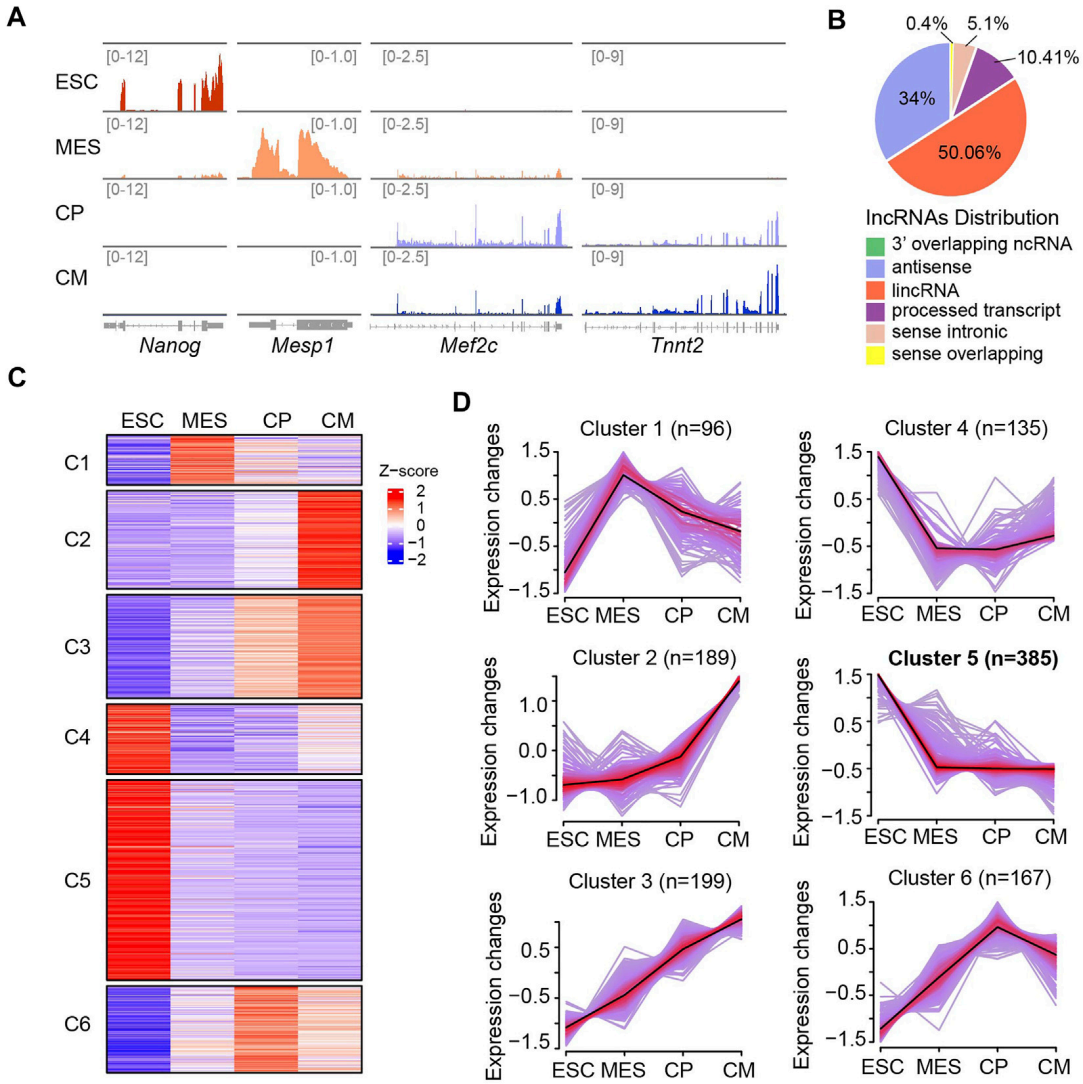
Dynamic lncRNA expression pattern during ESC-derived cardiac differentiation. **(A)** Integrative Genomics Viewer (IGV) of read coverage for specific marker genes at distinct differentiation phases including ESC (Day 0), MES (Day 4), CP (Day 6) and CM (Day 10). The number of reads indicate the peak heights in corresponding genomic regions. **(B)** Genomic annotation of lncRNAs (*n* = 6,061) showing that the majority of lncRNAs were mapped to lincRNA (50.06%) and antisense (34%). **(C)** Heat map showing the expression patterns of lncRNAs were grouped into six different groups (C1-C6) across four differentiation stages. **(D)** Clustering analysis showing the expression patterns of lncRNAs were divided into six different groups (C1-C6) as shown in **(C)**. The average trend is indicated by black lines. The number of genes is indicated for each cluster. The ESC-specific cluster (C5) contains the largest population of lncRNAs (*n* = 385).

To investigate the global expression profiles of lncRNA transcriptome during ESC-derived cardiac differentiation, we collected the samples at four different phases including undifferentiated ESC, mesoderm (MES), cardiac progenitor (CP), and CM. Purified RNA after removal of ribosomal RNA was utilized for sequencing. The reproducible data revealed that 18,749 mRNAs and 6,061 lncRNAs were expressed during cardiac differentiation (Supplementary Figure S1A). As stage-specific marker genes, *Nanog* and *Mesp1* were highly expressed at ESC and MES stages, while *Mef2c* and *Tnnt2* were expressed at higher levels in the CP and CM phases (Figure 1A), indicating the high effectiveness of lncRNA-seq datasets. Comparative analysis of the expression patterns of mRNAs and lncRNAs showed that the expression of lncRNAs was lower than that of mRNAs. Over 50% of mRNAs had TPM values > 20 at four distinct stages, whereas only less than 10% of lncRNAs had similar TPM values (Supplementary Figure S1B). Mapping of these lncRNAs to genomic regions revealed that about half of the lncRNAs were derived from intergenic regions (Figure 1B).

To detect expression changes of lncRNAs during ESC-derived cardiac differentiation, we performed Fuzzy C-means Clustering based on the TPM values. This analysis led to the identification of six different gene clusters with differential lncRNA expression patterns across the differentiation phases (Figures 1C,D). Cluster one and six contained a batch of lncRNAs that were specifically expressed at MES and CP stages, respectively. Both cluster two and three comprised genes that were specifically expressed at the CM stage. However, lncRNAs in cluster two showed mildly increased expression from the ESC to CP stage but dramatically upregulated expression from the CP to CM stage. In contrast, the expressions of lncRNAs in cluster three were increased gradually with the progression of cardiac differentiation (Figures 1C,D). LncRNAs in cluster four showed decreased expressions from the ESC to CP stage and slightly recovered expression at the CM stage, while cluster five contained largest population of lncRNAs (*n* = 385) which expressed at highest levels in ESC and considerably downregulated afterwards. Altogether, our results suggested lncRNA transcriptome underwent dynamic regulation during ESC-derived cardiac differentiation and identified a batch of potential non-coding regulators involved in cardiogenesis.

### Regulatory Properties and Cellular Functions of Embryonic Stem Cells-specific Long Noncoding RNAs

**FIGURE 2:**
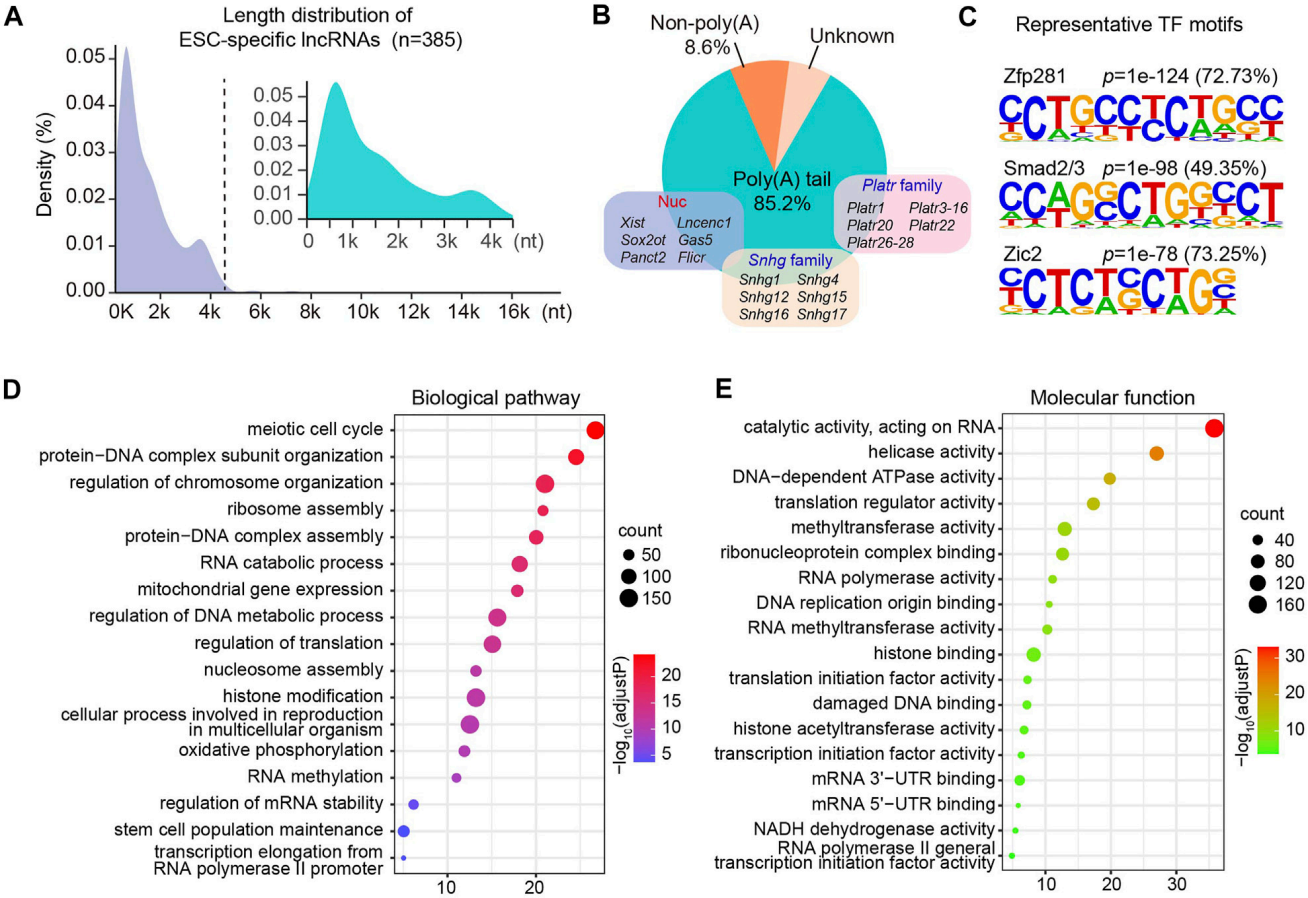
Regulatory features and cellular functions of ESC-specific lncRNAs. **(A)** Length distribution of ESC-specific lncRNA transcripts. **(B)** Polyadenylation features of ESC-specific lncRNAs. **(C)** Motif enrichment analysis showing representative TF motifs identified in the promoter region of genes coding ESC-specific lncRNAs. The selected motifs are linked to their corresponding TF. **(D,E)** Bubble plots show the biological pathways **(D)** and molecular functions **(E)** enriched by ESC-specific mRNAs co-expressed with lncRNAs. The size of a bubble is proportional to the number of genes. The intensity of the circle color is corresponding to the significance level which is indicated as -log_10_ (adjust *p*-value).

It has been well documented that pluripotent genes such as *Nanog* and *Sox2*, are restricted to pluripotent cells and downregulated upon differentiation (Zhao and Jin, 2017). Similar as the regulatory pattern of pluripotent genes, we identified that 385 lncRNAs were expressed specifically in ESC and downregulated upon differentiation. Close examination of these lncRNAs revealed that the majority of them were less than 4,000 nucleotides in length (Figure 2A). Furthermore, 85.2% of ESC-specific lncRNAs harbored poly(A) tails, which might be associated with their stabilization in the cellular compartment. As canonical non-coding regulators of cell fate in the nucleus, *Xist*, *Lncenc1*, and *Gas5*, were included in the subgroup of polyadenylated lncRNAs specifically expressed in ESC (Figure 2B), further suggesting the accuracy and validity of our lncRNA-seq data. Intriguingly, this cluster also contained a large number of members from two lncRNA families, *Platr* and *Snhg*, some of which have been reported to regulate the stem cell fate transition (Bergmann et al., 2015; Lu et al., 2019; Li et al., 2020; Du et al., 2021; Yan et al., 2021).

To understand the key regulators that control the gene expressions of these ESC-specific lncRNAs, we next assessed the TF-binding cis-elements imbedded in the promoter region of their coding genes. We observed that about 72.73% of the lncRNAs could be bounded by Zfp281 (Figure 2C), which has been reported to be not required for maintenance of ESCs but indispensable for proper differentiation by targeting core pluripotent genes (Fidalgo et al., 2011). In addition, Smad2/3 and Zic2 have been implicated in the self-renewal and ESC specification (Sakaki-Yumoto et al., 2013; Luo et al., 2015), and our analysis suggested that approximately 49.35 and 73.25% of ESC-specific lncRNAs were enriched for the Smad2/3 and Zic2 motif, respectively (Figure 2C).

The clustering analysis also led to identification of ten distinct expression patterns of protein-coding genes across four differentiation phases (Supplementary Figure S2). We then performed gene ontology (GO) enrichment analysis of ESC-specific protein-coding genes in cluster two that were co-expressed with specifically expressed lncRNAs in ESC. Our analysis revealed that they were significantly enriched in biological processes including meiotic cell cycle, protein-DNA complex assembly, ribosome assembly and protein translation. Interestingly, these co-expressed lncRNAs were also involved in RNA methylation and histone modification (Figure 2D). Consistent with their potential biological processes, these ESC-specific lncRNAs were co-expressed with genes encoding proteins with translation regulator activity, methyltransferase activity and histone acetyltransferase activity (Figure 2E). These results suggest that the ESC-retained lncRNAs are forming regulatory networks with stage-specific TFs and protein-coding genes to modulate ESC fate determination. Supporting this notion, a human ESC-specific lncRNA, *lncPRESS1*, has been reported to be not only directly regulated by transcription factor P53, but also interact with SIRT6 and modulate histone H3K56 and H3K9 acetylation to effect ESC state (Jain et al., 2016).

### Identification of Embryonic Stem Cell-specific Long Noncoding RNA-Binding Proteins

**FIGURE 3:**
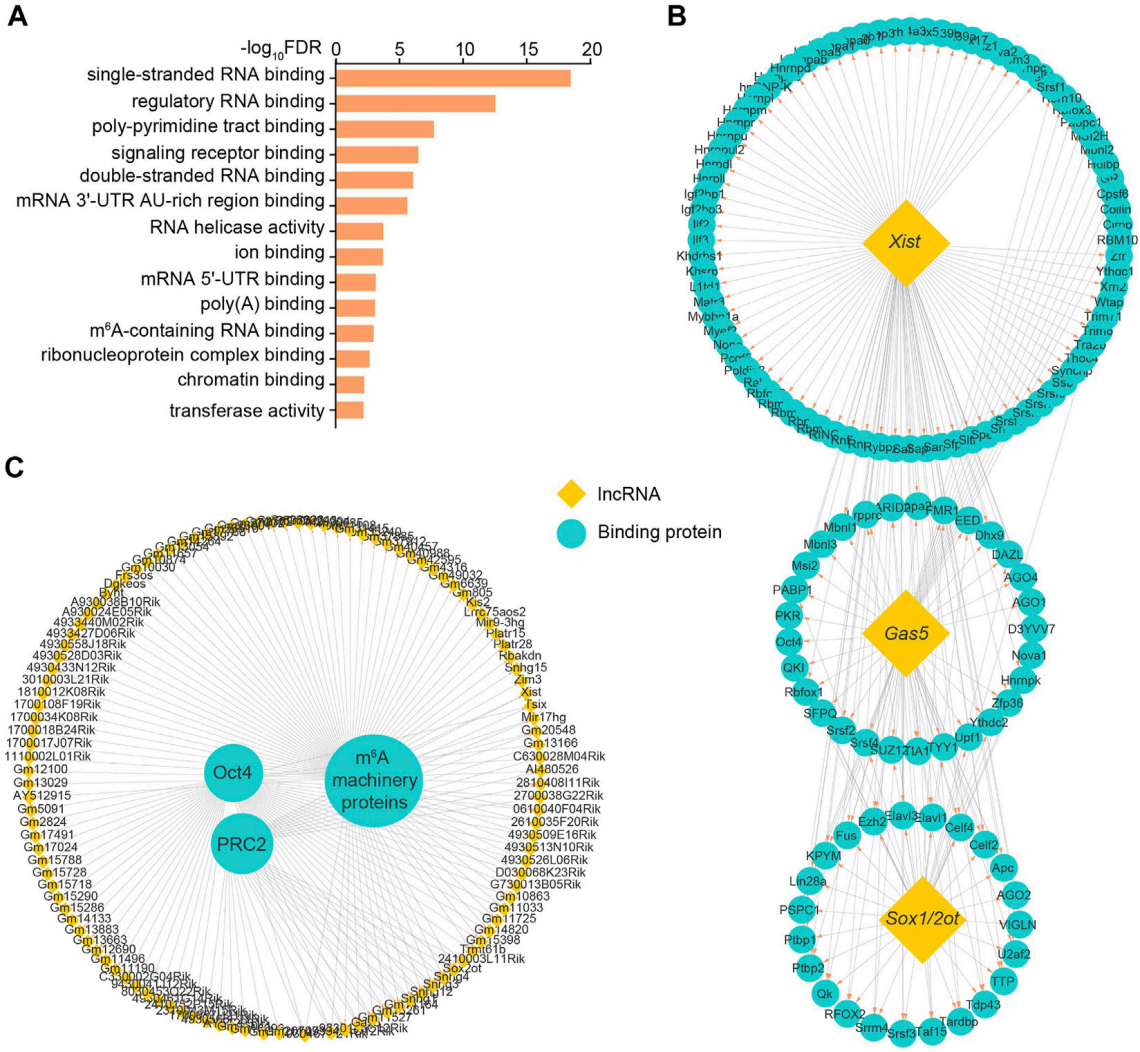
Identification of ESC-specific lncRNA-binding proteins. **(A)** GO analysis of molecular functions enriched by identified binding proteins for ESC-specific lncRNAs. **(B)** The lncRNA-protein interaction network identifies the candidate proteins binding with ESC-specific lncRNAs *Xist*, *Gas5* or *Sox1/2ot*. **(C)** The lncRNA-protein interaction network identifies the candidate lncRNAs binding with pluripotency regulators Oct4, PRC2 and m^6^A machinery proteins. Yellow and green respectively represent lncRNAs and the interacting proteins of lncRNAs. See also Supplementary Table S2.

LncRNA generally modulates target gene expression through recruiting specific functional proteins (Statello et al., 2021). We therefore searched for ESC-specific lncRNA-binding proteins by integrative analysis with two RNA-binding protein databases from both NPinter and RNAinter (Teng et al., 2020; Kang et al., 2022). Eventually we identified 1719 pairs of lncRNA-protein interactions. GO analysis of these binding proteins revealed that they harbored molecular activities of single- and double-stranded RNA binding, RNA helicase and chromatin binding. Surprisingly, m^6^A-containing RNA binding activity was also found in these lncRNA-interacting proteins (Figure 3A).

For individual lncRNAs, *Gas5* and *Sox1/2ot*, which are linked to the regulation of pluripotency and specific linage differentiation (Knauss et al., 2018; Tu et al., 2018; Xi et al., 2022), were able to recruit many functional factors, such as Ago and Srsf family proteins (Figure 3B). Consistent with multi-faceted roles reported previously (Plath et al., 2003; Fang et al., 2004; Patil et al., 2016), *Xist* lncRNA could guide the regulation of gene expression through coupling with a variety of proteins including the key components of PRC and m^6^A machineries (Figure 3B). Among the identified proteins, the most representative ones are the core pluripotency component Oct4, histone modification activator PRC2 and m^6^A machinery proteins such as Igf2bp1/2/3 and Ythdc1/2, which modulate lncRNA functions potentially through direct interaction (Figure 3C). These results suggest that these ESC-specific lncRNAs mediate multi-layered regulation of gene expression through transcription, epigenetic and epitranscriptomic modifications.

### Modification of Embryonic Stem Cell-specific Long Noncoding RNAs by m^6^A

**FIGURE 4:**
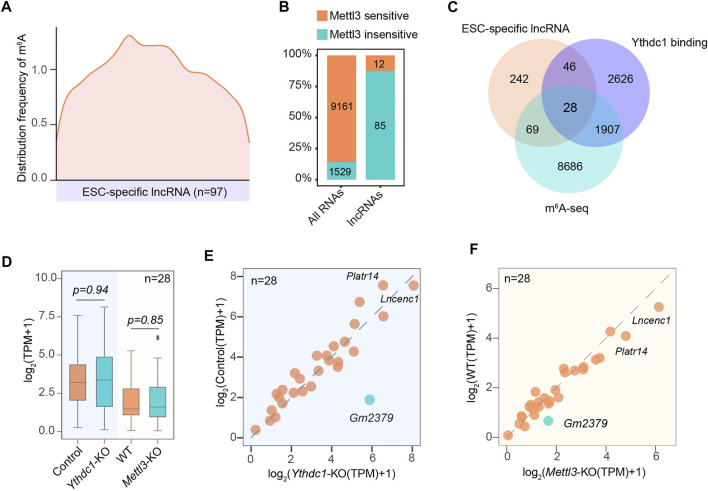
Characterization of the modification status on ESC-specific lncRNAs by m^6^A. **(A)** Transcriptome-wide distribution pattern of m^6^A for ESC-specific m^6^A-containing lncRNAs. **(B)** The percentage and number of ESC-specific lncRNAs containing m^6^A peaks that are regulated by Mettl3. **(C)** A Venn diagram shows the overlapping of lncRNA transcripts containing m^6^A sites and binding with nuclear m^6^A “reader” Ythdc1. **(D)** A box plot shows the expression of 28 overlapped lncRNAs **(C)** containing m^6^A sites and binding with Ythdc1 in response to the depletion of Mettl3 or Ythdc1. **(E)** A scatter plot showing the expression changes of 28 overlapped lncRNAs **(C)** in the presence or absence of Ythdc1. **(F)** A scatter plot showing the expression changes of 28 overlapped lncRNAs **(C)** in the presence or absence of Mettl3. *Gm2379* is highlighted in green.

Given that the ESC-specific lncRNAs are directly recognized by m^6^A machinery proteins, we therefore hypothesized that at least some of them could be modified by RNA methylation. Indeed, 97 out of 385 lncRNAs contained typical m^6^A peaks. Distinct from m^6^A distribution in the vicinity of the stop codons of mRNAs (Dominissini et al., 2012; Meyer et al., 2012), m^6^As in lncRNAs were highly enriched in close proximity to the middle of the transcript body (Figures 4A,C). In addition, over 85% of mRNA m^6^As were subject to the regulation of core methyltransferase Mettl3. While, only less than 13% of m^6^As in ESC-associated lncRNAs were regulated by Mettl3 (Figure 4B), suggesting the existence of additional methyltransferase catalyzing the m^6^A formation of these lncRNAs.

Since Ythdc1 has recently been identified to regulate the functionality of a number of non-coding RNAs (Wei and He, 2021), we next analyzed whether these m^6^A-containing lncRNAs could recruit Ythdc1 as the “reader” protein. As shown in Figure 4C, 28 out of 97 lncRNAs harbored m^6^A peaks that were targeted for regulation by Ythdc1 (Figure 4C). The remaining 69 lncRNAs may be regulated by other “reader” proteins. Surprisingly, although methylated adenosines of the 28 lncRNAs were directly bound by Ythdc1, almost all of them did not significantly change their expressions upon loss of Mettl3 or Ythdc1 (Figures 4D–F). A recent study has indicated that the RNA transcribed from long interspersed nuclear element-1 (LINE1) acts as a binding scaffold for Ythdc1 to regulate transcription activities in early embryo and mouse ESCs (Chen et al., 2021; Liu et al., 2021). It is possible that Ythdc1 may facilitate the formation of lncRNA-scaffold complex rather than regulate its abundance to modulate the nuclear gene expression in ESC. Intriguingly, one out of the 28 lncRNAs, *Gm2379*, was dually regulated by Mettl3 and Ythdc1, as loss of either m^6^A machinery protein resulted in obviously increased expression of *Gm2379* (Figures 4E,F), suggesting that m^6^A may form different regulatory mode with lncRNAs to modulate their functionality.

### Characterization of the Long Noncoding RNA *Gm2379*

**FIGURE 5:**
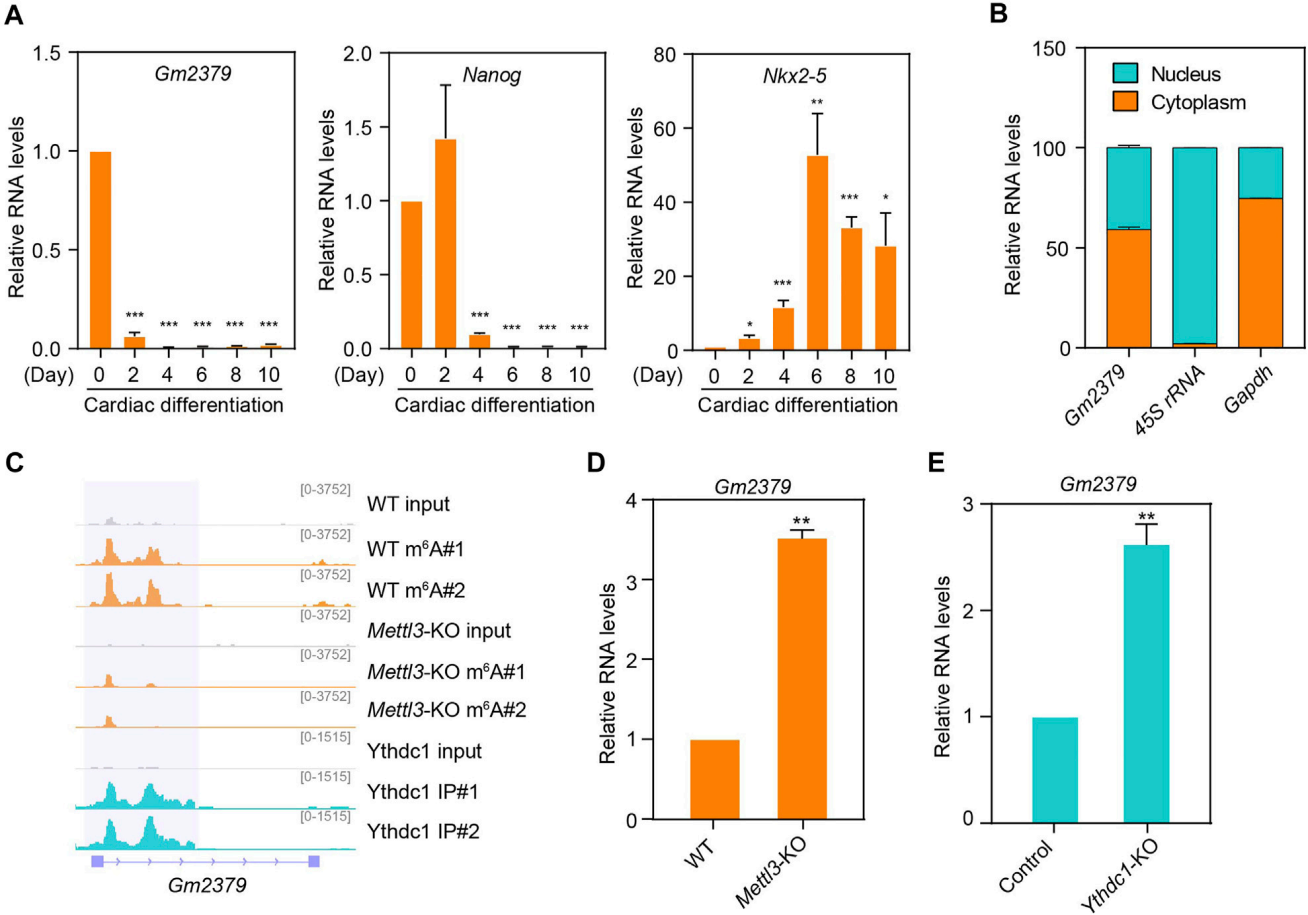
The m^6^A-harboring lncRNA *Gm2379* is regulated by both Mettl3 and Ythdc1. **(A)** Detection of *Gm2379* transcript levels during ESC-derived cardiac differentiation by RT-qPCR. *Nanog* and *Nkx2-5* serve as pluripotent and cardiac maker genes. Error bars, mean ± SEM; *n* = 4 independent replicates; *p < 0.05, **p < 0.01, ***p < 0.001. **(B)** Detection of the distribution of *Gm2379* transcript in ESCs by cell fractionation assay. The RT-qPCR data represented a percentage of the total amount of detected transcripts. Error bars, mean + SEM; *n* = 2 independent replicates. **(C)** Genome browser view of a representative genomic region showing m^6^A read density and Ythdc1-binding feature of the *Gm2379* transcript. **(D)** Detection of *Gm2379* transcript levels in WT and *Mettl3*-KO ESCs by RT-qPCR. Error bars, mean + SEM; *n* = 3 independent replicates; **p < 0.01. **(E)** Detection of *Gm2379* transcript levels in control and *Ythdc1*-KO ESCs by RT-qPCR. Error bars, mean + SEM; *n* = 4 independent replicates; **p < 0.01.

We next focused on the m^6^A-containing transcript *Gm2379*, as it is the only lncRNA that responds to the loss of the m^6^A machinery proteins. We initially validated the expression pattern of *Gm2379* during ESC-derived cardiac differentiation. As shown in Figure 5A, its expression was dramatically decreased at day 2 when embryonic bodies (EBs) were formed. Comparatively, the abundance of core pluripotency gene *Nanog* still maintained at high levels at this stage and declined after EBs generation. *Nkx2-5* as one of the earliest markers of the cardiac differentiation, was found to be obviously upregulated at day 6. To decipher the function of *Gm2379*, we next probed its expression in separated nuclear and cytoplasmic fractions from cultured ESCs. Different from the nuclear 45S rRNA, *Gm2379* was localized in both compartments (Figure 5B). Consistent with the global pattern of 97 ESC-specific lncRNAs, the two main m^6^A peaks of *Gm2379* were distributed in proximity to the middle of the transcript body as well as subject to the regulation of Mettl3 and Ythdc1, as m^6^A signals of *Gm2379* were overlapped with Ythdc1 RIP-seq signals and almost disappeared upon knockout of Mettl3 (Figure 5C). In addition, the transcript levels of *Gm2379* were significantly increased in ESCs depleting Mettl3 or Ythdc1 (Figures 5D,E), which is in good agreement with previous data analysis. Altogether, our findings indicate that *Gm2379* is specifically expressed in ESCs and dynamically regulated by nuclear “reader” protein Ythdc1 in a m^6^A-dependent manner.

### *Gm2379* Modulates the Expressions of Pluripotent and Germ-Layer Genes

**FIGURE 6:**
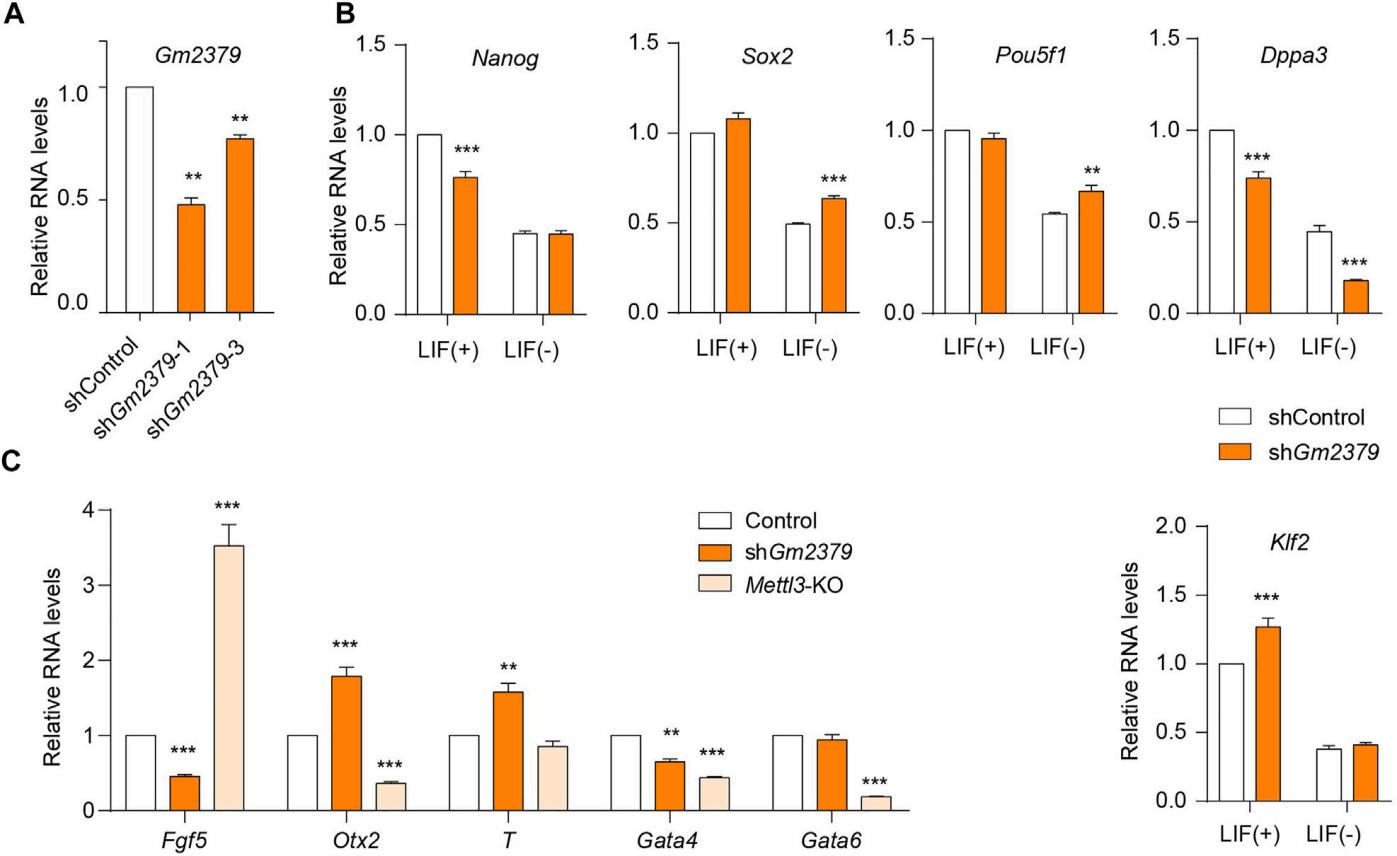
*Gm2379* modulates the expressions of pluripotent and germ-layer genes. **(A)** Generation of *Gm2379*-KD ESCs. **(B)** Detection of transcript levels of pluripotent genes in control and *Gm2379*-KD ESCs in the presence or absence of LIF by RT-qPCR. **(C)** Detection of transcript levels of three germ-layer genes in control, *Gm2379*-KD and *Mettl3*-KO ESCs by RT-qPCR. sh*Gm2379*-1 ESCs were used in **(B)** and **(C)**. Error bars, mean + SEM; *n* = 4 independent replicates; *p < 0.05, **p < 0.01, ***p < 0.001.

m^6^A has been implicated in the regulation of ESC self-renewal and differentiation. Depletion of Mettl3 results in m^6^A erasure on target pluripotent mRNAs, facilitating ESC self-renewal and impairing directed differentiation (Batista et al., 2014; Geula et al., 2015). The nuclear “reader” Ythdc1 has been found to be required for the maintenance of ESC pluripotency in a m^6^A-dependent manner (Chen et al., 2021; Liu et al., 2021). We have revealed that the expression of *Gm2379* lncRNA is regulated by both Mettl3 and Ythdc1, serving as a potential target of m^6^A in ESC. To determine whether *Gm2379* is directly involved in the regulation of pluripotency, we generated stable ESC lines with shRNA-mediated gene knockdown of *Gm2379* (Figure 6A). LIF is indispensable to maintain the pluripotent state of ESC (Martello and Smith, 2014). In the presence of LIF, depletion of *Gm2379* selectively affected the expression of pluripotency genes. The transcript levels of *Nanog* and *Dppa3* were significantly decreased in *Gm2379-*knock down (KD) cells, while *Klf2* was upregulated in *Gm2379*-KD cells (Figure 6B). However, in the absence of LIF, the expression of *Nanog* and *Klf2* rather than *Dppa3* was comparable in control and *Gm2379*-KD ESCs, suggesting the selective regulation of pluripotency genes by *Gm2379* lncRNA in a LIF-dependent manner.

In addition to the pluripotent genes, the crucial genes involved in the formation of three germ layers (ectoderm, mesoderm and endoderm) were dramatically dysregulated in *Gm2379*-KD ESCs (Figure 6C)*.* Specifically, the ectoderm gene *Fgf5* and endodermal gene *Gata4* were significantly downregulated while the transcripts of the mesoderm gene *T* and ectoderm gene *Otx2* were obviously increased in response to *Gm2379* depletion. Mettl3, as the core m^6^A methyltransferase, has been demonstrated to regulate ESC pluripotency and differentiation (Batista et al., 2014; Geula et al., 2015). In contrast to *Gm2379* KD, loss of Mettl3 resulted in increased expression of *Fgf5* and decreased expression of *Otx2* (Figure 6C). Considering the negative regulation of *Gm2379* by Mettl3 shown above, it is very possible that Mettl3 directly targets *Gm2379* lncRNA to modulate the expression of ectodermal genes.

## Discussion

LncRNA as genomic “dark matter” has been implicated to play crucial roles in multiple cellular processes by forming signaling regulatory networks with other functional molecules. In the process of cell fate regulation, only a small fraction of lncRNAs has been characterized (Fico et al., 2019; Chen et al., 2020). Therefore, it could be expected that many more non-coding transcripts will participate in the regulation of pluripotency and differentiation processes. In this study, we performed a global investigation of lncRNA expression profiles across four different phases of ESC-derived cardiac differentiation. Our findings showed that the lncRNA transcriptome changed dramatically during cardiac differentiation (Figures 1C,D), revealing the dynamic and complex regulation of cell differentiation at additional layers.

Among the six lncRNAs clusters with distinct expression patterns we identified, the cluster five contained the largest number of non-coding transcripts which expressed at most abundant levels in ESC and dramatically downregulated at MES stage and subsequent phases during cardiac differentiation (Figures 1C,D), suggesting the crucial roles of lncRNA in the regulation of ESC gene expression. Careful inspection revealed that many reported lncRNAs in the regulation of cell fate were included in this subgroup, such as *Gas5* and *Lncenc1*. Depletion of *Gas5* has been found to dysregulate a number of critical genes involved in self-renewal and endodermal differentiation through forming interaction networks with pluripotent transcriptional factors and epigenetic factors (Tu et al., 2018). *Lncenc1* maintains the self-renewal of naive ESCs through cooperation with PTBP1 and HNRNPK to regulate the expression of glycolytic genes (Sun et al., 2018). Therefore, it is reasonable to speculate that the highly specific expression of lncRNAs in ESC is tightly associated with their molecular functions in the regulation of pluripotency and differentiation. In addition, our analysis also revealed a number of previously unappreciated transcripts from same lncRNA families (Figure 2B), which will facilitate our understanding of dynamics and complexity of lncRNA regulatory networks governing cell fate determination.

The function of lncRNAs is accurately exerted dependent on the formation of both cis-regulatory modules with TFs and trans-regulatory modules with RBPs (Statello et al., 2021). An increasing number of studies have been absorbed in deciphering how lncRNA regulates target gene expression, while few researches investigate how lncRNA itself is transcriptionally regulated (Tompkins et al., 2016). Consistent with the stage-specific transcriptional features, our analytic data showed that a large portion of ESC-specific lncRNAs were directly bounded and regulated by the pluripotency TF regulators Zfp281, Smad2/3 and Zic2 (Figure 2C). In addition, a number of these non-coding transcripts were found to cooperate with stage-specific trans RBPs in the regulation of ESC gene expression, such as Oct4 and PRC2 (Figure 3C). Surprisingly, m^6^A machinery proteins were also identified as direct interaction partners of ESC-specific lncRNAs. These observations collectively suggest that the ESC-retained lncRNAs exhibit subtle signaling networks with crucial TF regulator and trans RBPs to modulate cell pluripotency and differentiation progression.

Considering the m^6^A machineries identified as representative lncRNA-binding proteins, we thus evaluated the methylation status of these ESC-specific lncRNAs. Approximately 25% of these non-coding transcripts were modified by m^6^A. Whereas, only a small portion of m^6^A-containing lncRNAs were subject to the regulation of m^6^A-catalyzer Mettl3 (Figure 4B). Consistent with this observation, a recent study also found a subgroup of chromatin-associated LINE1 regulatory RNAs harbored m^6^A peaks that are insensitive to Mettl3 (Chen et al., 2021). It is possible that other methyltransferases such as Mettl16, mediate m^6^A formation for those Mettl3-insensitive ESC-specific lncRNAs. An increasing number of studies have been identified Ythdc1 as a primary m^6^A “reader” to regulate molecular functions of non-coding transcripts (Patil et al., 2016; Liu et al., 2020, 2021; Chen et al., 2021; Ji et al., 2021; Lee et al., 2021; Ninomiya et al., 2021; Wang et al., 2021). However, our survey revealed that only about 28% of m^6^A-containing lncRNAs were directly targeted by Ythdc1. More interestingly, only one out of the 28 Ythdc1-binding m^6^A-containing lncRNAs, *Gm2379*, dramatically altered its expression in response to the deletion of Mettl3 or Ythdc1 (Figures 4E,F), suggesting that majority of these non-coding transcripts may act as dynamic structural scaffolds for recruiting Ythdc1, which in turn does not regulate their expressions.

We next focused on the characterization of the novel lncRNA *Gm2379*. Consistent with the lncRNA-seq data, the m^6^A-containing lncRNA *Gm2379* was dramatically downregulated during ESC-derived cardiac differentiation (Figures 5A,C). Furthermore, depletion of Mettl3 or Ythdc1 leads to significantly increased expression of *Gm2379* (Figures 5D,E). In addition to the nuclear “reader” Ythdc1, it is very likely subject to the regulation of additional m^6^A-binding proteins in the cytoplasm, as *Gm2379* was found in both nuclear and cytoplasmic compartments (Figure 5B). Functional identification showed that depletion of *Gm2379* resulted in limited but significant alteration of pluripotent gene expressions. Instead, the key genes required for the formation of three germ layers, were more dramatically dysregulated upon knockdown of *Gm2379* (Figure 6). The opposite regulation of ectodermal genes by Mettl3 and *Gm2379* suggest that *Gm2379* lncRNA might function as a direct target of Mettl3 to govern the formation of ectoderm. Further investigation should focus on the examination of additional cooperating factors that are required for *Gm2379*-mediated ESC differentiation.

In summary, our findings provide global identification and characterization of lncRNA expression profiles during ESC differentiation. Investigation of cis and trans regulatory network suggest that ESC-specific lncRNAs are governed by key TFs as well as m^6^A effector machineries. Subsequent identification of a specific m^6^A-containing lncRNA on the molecular level further demonstrates the crucial interplay between epitranscriptomic modification and key lncRNAs in the regulation of cell state maintenance and transition.

## Data Availability

The datasets presented in this study can be found in online repositories. The names of the repository/repositories and accession number(s) can be found below: GEO, accession number GSE197205 (https://www.ncbi.nlm.nih.gov/geo/query/acc.cgi?acc=GSE197205).
