# Supplementary material for: Dynamic Transcriptome Profiling Reveals LncRNA-Centred Regulatory Networks in the Modulation of Pluripotency
Source: Front Cell Dev Biol. 2022 May 11;10:880674. doi: 10.3389/fcell.2022.880674 (PMC9130768; doi:10.3389/fcell.2022.880674)
Supplement: Supplementary file 1 [file DataSheet1.PDF]

Supplementary Information

Dynamic Transcriptome Profiling Reveals LncRNA-centred Regulatory Networks in the Modulation of Pluripotency

Shen Wang[1†], Jun Zhang[1†], Yu'an Ding[1], Haotian Zhang[1], Xiang Wu[1], Lingci Huang[1], Junjie He[1], Jun Zhou[1*], Xiao-Min Liu[1*]

[1]School of Life Science and Technology, China Pharmaceutical University, Nanjing, Jiangsu 210009, China

[2]Key Laboratory of Pathogen Biology of Jiangsu Province, Nanjing, China

[†]These authors have contributed equally to this work.

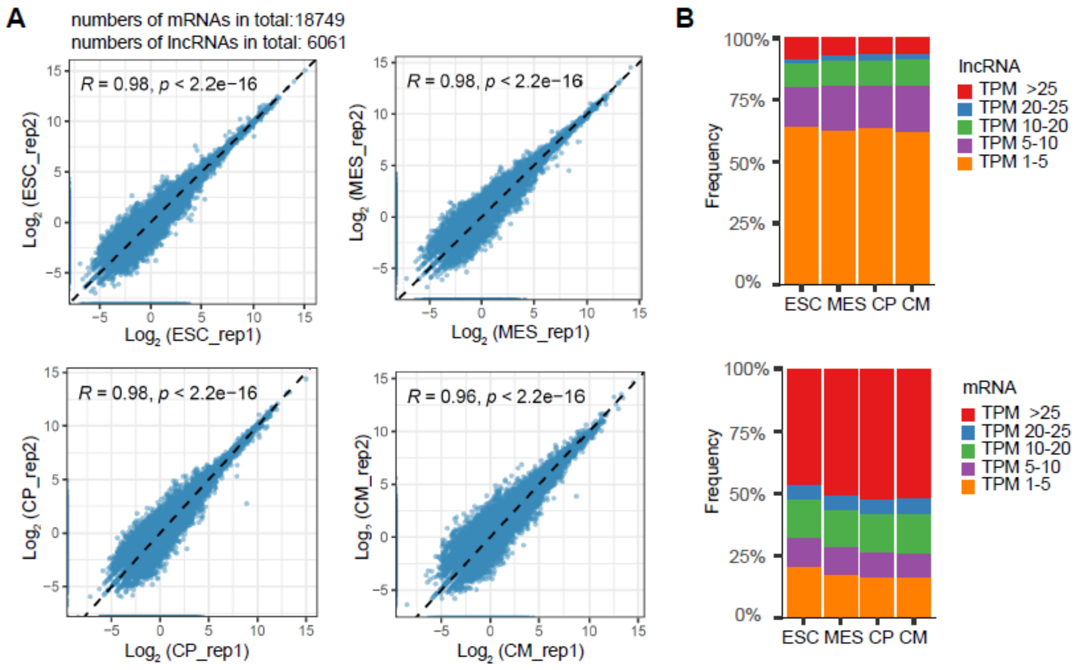

FIGURE S1 Dynamic lncRNA expression during ESC-derived cardiac differentiation. (A) Correlation scatterplots showing the reproducibility of RNA-seq replicates at each differentiation stage. Pearson's correlation is indicated at the top of each plot. (B) Distribution of different abundances of lncRNAs and mRNAs at each differentiation stage. Compared with mRNAs, the majority of lncRNAs were expressed at lower levels.

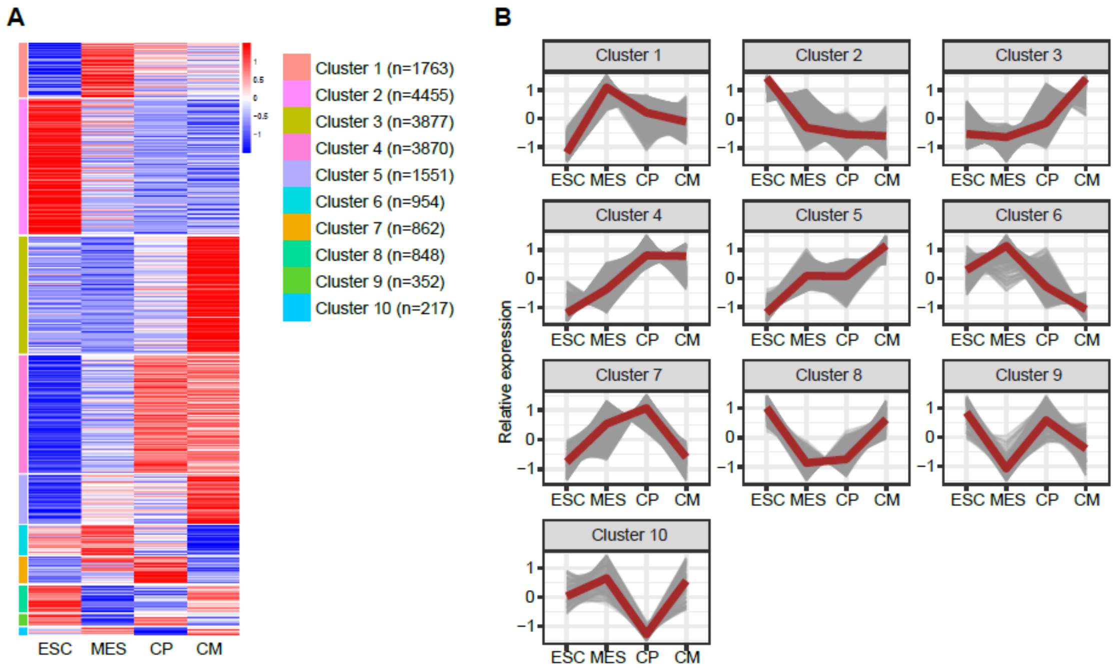

FIGURE S2 Dynamic mRNA expression pattern during ESC-derived cardiac differentiation. (A) Heat map showing the expression patterns of mRNAs were grouped into ten different groups across four differentiation stages. (B) Clustering analysis showing the expression patterns of mRNAs were divided into ten different groups as shown in (A). The average trend is indicated by red lines.

TABLE S1 Primers for qPCR used in this study.

Gene name	Forward	Reverse
Gm2379	5′-AGGCACGGCATTACAGAATT-3′	5′-GACATTCATCTCCACACTGG-3′
Nanog	5′-CAGCATCCATTGCAGCTATCC-3′	5′-CTGCCCCACATGGAAAGG-3′
Nkx2-5	5′-CTTTAGGAGAAGGGCGATGAC-3′	5′-AGGCTACGTCAATAAAGTGGG-3′
β-actin	5′-TTGCTGACAGGATGCAGAAG-3′	5′-ACTCCTGCTTGCTGATCCACAT-3′
Gapdh	5′-AATGTGTCCGTCGTGGATCT -3′	5′-AGACAACCTGGTCCTCAGTG-3′
45S rRNA	5′-TTCTCTTGTTCTGTGTCTGCC-3′	5′-GGGAGAAACAAGCGAGATAGG-3′
Pou5f1	5′- TCGGACCAGGCTCAGAGGTA-3′	5′- ATCCCTCCGCAGAACTCGTA-3′
Sox2	5′-AAACCAAGACGCTCATGAAGAAG-3′	5′-CGCTCGCCATGCTGTTC-3′
Fgf5	5′-GGGATTGTAGGAATACGAGGAGTT-3′	5′-TGGCACTTGCATGGAGTTT-3′
Otx2	5′-GGAGAGGACGACATTTACTAGG -3′	5′-TTCTGACCTCCATTCTGCTG-3′
T	5′-TCCCGAGACCCAGTTCATAG-3′	5′-TTCTTTGGCATCAAGGAAGG-3′
Gata4	5′-CACTTAGGGATATGGGTGTTCC-3′	5′-GGCAGGTGGAGAATAAGGAAG-3′
Gata6	5′-TTCTACACAAGCGACCACCT-3′	5′-CACCAAGAATCCTGTCGCAC-3′

TABLE S2 List of lncRNA-protein interactions analyzed based on NPinter and RNAinter databases.

Detailed interaction list for Figure 3B:

lncRNA name	Protein name
Xist	Apc Celf1 Celf2 Celf4 Mbnl1 Msi2 Ptbp1 Ptbp2 Rbfox1 Srrm4 Tardbp U2af2 Zfp36 Hnrnpc Lin28a Rbm3 Hnrnpk Elavl1 Dhx9 Taf15 Nova2 Mbnl3 Qk Hnrnpa2b1 Nova1 Elavl3 Celf1 Ciz1 Ddx17 Ddx39a Ddx39b Ddx5 Dhx9 Eif4a3 Elavl1 Erh Fubp3 Fus Hmgb1 Hnrnpa0 Hnrnpa1 Hnrnpa2b1 Hnrnpa3 Hnrnpab Hnrnpc Hnrnpd Hnrnph3 HnRNP-K Hnrnpl Hnrnpm Hnrnpr Hnrnpu Hnrnpul2 Hnrpdl Hnrpll Igf2bp1 Igf2bp3 Ilf2 Ilf3 Khdrbs1 Khsrp L1td1 Lin28a Matr3 Mybbp1a Myef2 Nono Pcgf5 Poldip3 Ptbp1 Ptbp2 Raly Rbfox2 Rbm14 Rbm15 Rbm3 Rbm4 Rbmxl1 RING1B Rnf20 Rnmt Rybp Safb Safb2 Sap18 Sarnp Sfpq Sltm Spen Srrt Srsf10 Srsf2 Srsf3 Srsf5 Srsf7 Srsf9 Ssb Syncrip Tardbp Thoc4 Tra2b Trim6 Trim71 Wtap Xrn2 Ythdc1 Zfr Nova1 PKR Ezh2 Tdp43 PSPC1 RFOX2 AGO1 AGO2 AGO4 TTP D3YVV7 TAF15 VIGLN TIA1 SFPQ KPYM CIZ1
Gas5	AGO1 AGO2 AGO4 Apc Celf2 Celf4 Cirbp Coilin Cpsf6 Cpsf6 DAZL Dhx9 EED Elavl1 Elavl3 Ezh2 FMR1 Fus GR Hdlbp Hnrnpa2b1 JARID2 KPYM Lin28a Lrpprc Mbnl1 Mbnl1 Mbnl2 Mbnl2 Mbnl3 Mbnl3 Msi2 MSI2H PABP1 Pabpc1 PKR Pou5f1 PSPC1 Ptbp1 Ptbp2 Ptbp2 Qk QKI Rbfox1 Rbfox3 Rbfox3 Rbfox3 Rbm10 RBM10 RFOX2 SFPQ Srrm4 Srsf1 Srsf2 Srsf3 Srsf4 SUZ12 Taf15 Tardbp Tdp43 TIA1 TTP TYY1 U2af2 Upf1 VIGLN Ythdc2 Zfp36
Sox1/2-ot	Fus Srsf3 Tardbp U2af2 Hnrnpk Elavl3 Apc Celf2 Celf4 Lin28a Ptbp1 Ptbp2 Srrm4 Srsf3 Srsf4 Taf15 Tardbp U2af2 Upf1 Ythdc2 Lrpprc Qk Upf1 JARID2 Ezh2 Tdp43 QKI PSPC1 FMR1 DAZL AGO2 RFOX2 TTP Elavl1 EED SUZ12 OCT4 TYY1 Nova1 D3YVV7 PABP1 TAF15 VIGLN Ptbp2 RBM10 SFPQ KPYM

Detailed interaction list for Figure 3C:

Protein name	lncRNA name
Oct4	0610040F04Rik 1700057H21Rik 2310043M15Rik 2410003L11Rik 2410152P15Rik 2610035F20Rik 2700038G22Rik 4930461G14Rik 4930467D21Rik 4930509E16Rik 4930513N10Rik 4930526L06Rik 4933404O12Rik 8030453O22Rik 9330185C12Rik 9430041J12Rik C330002G04Rik C630028M04Rik D030068K23Rik G730013B05Rik Gas5 Gm10863 Gm11033 Gm11190 Gm11496 Gm11527 Gm11725 Gm12690 Gm13166 Gm13261 Gm13663 Gm13883 Gm14133 Gm14164 Gm14820 Gm15286 Gm15290 Gm15398 Gm15718 Gm15728 Gm15788 Gm17024 Gm17491 Gm2824 Gm5091 Snhg1 Snhg12 Snhg3 Snhg4 Sox2ot Trmt61b Tsix
PRC2 (Ezh2, Eed, Suz12 and Jarid2)	2410003L11Rik 2810408I11Rik 4933404O12Rik C630028M04Rik Gas5 Gm14164 Gm16041 Gm20548 Mir17hg Snhg12 Sox2ot Tsix Xist 4933404O12Rik 9330185C12Rik Snhg1 Snhg3 2700038G22Rik 4930467D21Rik 4933404O12Rik 9330185C12Rik AY512931 Gm11527 Snhg4 2410003L11Rik 4930566F21Rik 4933404O12Rik AI480526 Gm13166 Gm13261 Gm14164 Gm16096 Gm16211 Gm20707
m^{6}A machinery proteins (Fmr1, Hnrnpa2b1, Rbm15, Wtap, Igf2bp1/2/3 and Ythdc1/2)	4930467D21Rik 4933404O12Rik 9330185C12Rik AY512915 D030068K23Rik G730013B05Rik Gas5 Gm10863 Gm11033 Gm13029 Gm14164 Gm14820 Mir17hg Snhg1 Snhg12 Sox2ot Trmt61b Gas5 Gm12100 Xist Gm12100 0610040F04Rik 1110002L01Rik 1700017J07Rik 1700018B24Rik 1700034K08Rik 1700108F19Rik 1810012K08Rik 2610035F20Rik 2810408I11Rik 3010003L21Rik 4930433N12Rik 4930467D21Rik 4930509E16Rik 4930513N10Rik 4930526L06Rik 4930528D03Rik 4930558J18Rik 4933404O12Rik 4933427D06Rik 4933440M02Rik 9330185C12Rik A930024E05Rik A930038B10Rik AI480526 Bvht Dgkeos Frs3os G730013B05Rik Gm10030 Gm10874 Gm11527 Gm11657 Gm11725 Gm12100 Gm13054 Gm13261 Gm14264 Gm15398 Gm15892 Gm17768 Gm20548 Gm26510 Gm26901 Gm27002 Gm2762 Gm28373 Gm28626 Gm29243 Gm29994 Gm30085 Gm31108 Gm31415 Gm35240 Gm37885 Gm37912 Gm40457 Gm40988 Gm42595 Gm4316 Gm49032 Gm6639 Gm805 Kis2 Lrrc75aos2 Mir17hg Mir9-3hg Platr15 Platr28 Rbakdn Snhg15 Snhg3 Snhg4 Sox2ot Trmt61b Zim3
